# Opicapone versus placebo in the treatment of Parkinson’s disease patients with end-of-dose motor fluctuation-associated pain: rationale and design of the randomised, double-blind OCEAN (OpiCapone Effect on motor fluctuations and pAiN) trial

**DOI:** 10.1186/s12883-022-02602-8

**Published:** 2022-03-12

**Authors:** K. Ray Chaudhuri, Per Odin, Joaquim J. Ferreira, Angelo Antonini, Olivier Rascol, Mónica M. Kurtis, Alexander Storch, Kirsty Bannister, Patrício Soares-da-Silva, Raquel Costa, Diogo Magalhães, José Francisco Rocha

**Affiliations:** 1 King’s College London, Department of Neurosciences, Institute of Psychiatry, Psychology & Neuroscience and Parkinson’s Foundation Centre of Excellence, King’s College Hospital, London, UK; 2 Department of Neurology, Lund University, Lund, Sweden; 3 Laboratory of Clinical Pharmacology and Therapeutics, Faculdade de Medicina, Universidade de Lisboa, Lisbon, Portugal; 4 Parkinson Disease and Movement Disorder Unit, Department of Neurosciences, University of Padova, Padova, Italy; 5 Toulouse Parkinson Expert Center, Departments of Neurosciences and Clinical Pharmacology, Centre d’Investigation Clinique de Toulouse CIC1436, NS-Park/FCRIN Network, and NeuroToul COEN Center, University Hospital of Toulouse, INSERM, University of Toulouse 3, Toulouse, France; 6 Movement Disorders Unit, Neurology Department, Hospital Ruber Internacional, Madrid, Spain; 7 Department of Neurology, University Medical Center Rostock, and German Center for Neurodegenerative Diseases (DZNE), Rostock, Germany; 8 Institute of Psychiatry, Psychology and Neuroscience, King’s College London, London, UK; 9 BIAL – Portela & Ca S.A., Coronado, Portugal; 10 MedInUP-Center for Drug Discovery and Innovative Medicines, University of Porto, Porto, Portugal

**Keywords:** Dopamine, King’s Parkinson’s disease Pain Scale, Levodopa, Motor fluctuations, Non-motor fluctuations, Non-motor symptoms, Opicapone, Pain, Parkinson’s disease

## Abstract

**Background:**

Optimisation of dopaminergic therapy may alleviate fluctuation-related pain in Parkinson’s disease (PD). Opicapone (OPC) is a third-generation, once-daily catechol-O-methyltransferase inhibitor shown to be generally well tolerated and efficacious in reducing OFF-time in two pivotal trials in patients with PD and end-of-dose motor fluctuations. The OpiCapone Effect on motor fluctuations and pAiN (OCEAN) trial aims to investigate the efficacy of OPC 50 mg in PD patients with end-of-dose motor fluctuations and associated pain, when administered as adjunctive therapy to existing treatment with levodopa/dopa decarboxylase inhibitor (DDCi).

**Methods:**

OCEAN is a Phase IV, international, multicentre, randomised, double-blind, placebo-controlled, parallel-group, interventional trial in PD patients with end-of-dose motor fluctuations and associated pain. It consists of a 1-week screening period, 24-week double-blind treatment period and 2-week follow-up period. Eligible patients will be randomised 1:1 to OPC 50 mg or placebo once daily while continuing current treatment with levodopa/DDCi and other chronic, stable anti-PD and/or analgesic treatments. The primary efficacy endpoint is change from baseline in Domain 3 (fluctuation-related pain) of the King’s Parkinson’s disease Pain Scale (KPPS). The key secondary efficacy endpoint is change from baseline in Domain B (anxiety) of the Movement Disorder Society-sponsored Non-Motor rating Scale (MDS-NMS). Additional secondary efficacy assessments include other domains and total scores of the KPPS and MDS-NMS, the Parkinson’s Disease Questionnaire (PDQ-8), the MDS-sponsored Unified Parkinson’s Disease Rating Scale (MDS-UPDRS) Parts III and IV, Clinical and Patient’s Global Impressions of Change, and change in functional status via Hauser’s diary. Safety assessments include the incidence of treatment-emergent adverse events. The study will be conducted in approximately 140 patients from 50 clinical sites in Germany, Italy, Portugal, Spain and the United Kingdom. Recruitment started in February 2021 and the last patient is expected to complete the study by late 2022.

**Discussion:**

The OCEAN trial will help determine whether the use of adjunctive OPC 50 mg treatment can improve fluctuation-associated pain in PD patients with end-of-dose motor fluctuations. The robust design of OCEAN will address the current lack of reliable evidence for dopaminergic-based therapy in the treatment of PD-associated pain.

**Trial registration:**

EudraCT number 2020–001175-32; registered on 2020-08-07.

## Background

Although fragmented accounts of Parkinsonism date back to 2500 BC, initial descriptions of its cardinal motor signs were much more recent (as in the 1690 book, ‘*Pax corporis*’, by Ferenc Pápai Páriz); however, it was only in 1817 that James Parkinson medically described the motor symptoms of the disease in such detail that the condition would subsequently be named after him [1–3].

Although the relationship between motor symptoms and non-motor symptoms (NMS) is variable and not necessarily linear, many NMS – specifically pain – can undergo fluctuations based on ON and OFF states during long-term treatment with levodopa [4, 5]. NMS that fluctuate in parallel with motor symptoms and in relationship to plasma levodopa levels have been termed non-motor fluctuations (NMF) [6] and encompass a range of neuropsychiatric (e.g. depression, apathy, fatigue), autonomic (e.g. sweating, micturition frequency/urgency), cognitive and sensory (e.g. pain) manifestations [5, 7, 8]. NMF have been reported to occur in 60–100% of patients with motor fluctuations, and may result in greater disability and burden than motor disturbances [5, 7]. NMF are complex, their appearance not always matching that of motor fluctuations in terms of timing [4, 8], and their underlying pathogenic mechanisms are still relatively unclear; however, sizable evidence suggests that, similarly to motor fluctuations, involvement of the dopaminergic system is key, with dopamine either being directly involved or working as a modulator of serotonin, norepinephrine or acetylcholine [7, 8]. In contrast to the relatively linear progression of most motor features during the disease course of PD, some NMS increase in frequency while others improve as dopaminergic therapy is initiated [8–10]; NMF are among those usually responsive to dopaminergic therapy optimisation [4, 7].

Pain is one of the most frequent and burdensome NMS in PD, being a significant comorbidity in up to 85% of PD patients, and may precede motor symptoms of the disease [11–16]. The pathophysiology underlying pain in PD is complex and not completely elucidated, and its management remains a key unmet need. The types and distribution of pain experienced by patients with PD are heterogeneous (Table 1) [17–20]. Spontaneous pain may be triggered by disease-related and/or comorbid conditions, exacerbated by a lowered pain threshold that may result from dysfunctional nociceptive processing caused by specific neurodegenerative changes [12, 21, 22]. Abnormal basal ganglia function in PD modulates pain directly and indirectly via mechanisms that impact both affective and cognitive nociceptive processing [23]. Pain has been shown to be associated with sleep disruption and cardiovascular disturbances in PD, and there is an indication that pain, sleep disruption and dysautonomia may share a common pathophysiology involving non-dopaminergic pathways [16]. Several recent publications have reviewed the current treatment options for each type of pain in PD [12, 19, 24, 25].

**Table 1:** Types of pain and specific features

Type of pain	Features (Ford 2010, Valkovic 2015, Edinoff 2020)
Musculoskeletal pain	• Aching, cramping, arthralgic, myalgic sensations in joints and muscles • May include muscle tenderness, arthritic changes, skeletal deformity, limited joint mobility, postural abnormalities, and antalgic gait • May be exacerbated by parkinsonian rigidity, stiffness and immobility, and alleviated by mobility • May fluctuate with medication dose and improves with levodopa
Radicular or peripheral neuropathic pain	• Pain in root or nerve territory, associated with motor or sensory signs of nerve or root entrapment
Dystonia-related pain	• Associated with sustained twisting movements and postures; muscular contractions often very forceful and painful • May fluctuate closely with medication dosing: early morning dystonia, OFF dystonia, beginning-of-dose and end-of-dose dystonia, peak dose dystonia
Primary/central pain	• Burning, tingling, formication, ‘neuropathic’ sensations; often relentless and bizarre in quality, not confined to root or nerve territory • Pain may have an autonomic character, with visceral sensations or dyspnoea, and vary in parallel with the medication cycle as NMF • Not explained by rigidity, dystonia, musculoskeletal or internal lesion
Akathitic discomfort/other pain	• Primary headache, visceral, arthritic, non-radicular low back pain, oral and genital pain • Unpleasant agitating sensation associated with restless legs syndrome

*NMF* non-motor fluctuations, *PD* Parkinson’s disease

Pain in PD is also associated with motor fluctuations [11, 26]. The role of dopamine in pain signalling is complex. Pain relief elicits rewards mediated by elevated dopamine in the nucleus accumbens, and reciprocity with higher brain regions such as the anterior cingulate cortex and dopaminergic transmission therein is necessary for the relief of pain aversiveness [27]. Meanwhile, along with serotonin and noradrenaline, dopamine may modulate pre-synaptic inhibition in the mouse spinal cord [28]. The precise mechanism underlying dopamine’s role in pain modulation is hitherto equivocal. Clinically, dopaminergic therapies have been shown to alleviate pain in PD [12, 21, 22, 29] and fluctuation-related pain in PD is believed to be partially mediated by dopamine [12, 30]. Studies that manipulate dopamine with the aim of translation to clinical therapy will be hampered by dopamine’s role in movement and reward [31]. While optimisation of dopaminergic therapy may alleviate fluctuation-related pain [12, 22, 32, 33], high-quality evidence of the benefit of dopaminergic therapies in PD-associated pain is lacking [30], with only one study coming close to providing Level 1 evidence, although failing to meet its primary endpoint at 16 weeks [34]. One reason for this is that, until recently, there were no disease-specific scales to adequately measure the heterogeneous types of pain in PD; this has now been resolved with the development and validation of the King’s Parkinson’s disease Pain Scale (KPPS) [35].

Levodopa is still the most effective symptomatic treatment for PD [36]. However, following oral administration, levodopa is extensively metabolised in the periphery by dopa decarboxylase (DDC) and catechol-O-methyltransferase (COMT), and only 1% of an oral dose of levodopa reaches the brain [37, 38]. Moreover, long-term treatment with levodopa is complicated by the development of wearing off and drug-induced dyskinesia [36, 39]. Pain increases during OFF periods and patients with dyskinesia have increased pain sensitivity [40, 41]. Treatment with levodopa has been shown to improve pain thresholds in patients with PD, unlike the dopamine agonist apomorphine [16, 42]. Inhibitors of DDC (DDCi) and COMT (COMTi) are commonly used as an adjunct to levodopa in patients with PD in order to increase levodopa bioavailability and its delivery to the brain, and thereby ameliorate wearing-off symptoms [38, 43, 44].

Opicapone (OPC) is a third-generation, once-daily COMTi [37, 38, 45, 46], which has been shown to be generally well tolerated and efficacious in reducing OFF-time in two pivotal trials in patients with PD and end-of-dose motor fluctuations (BIPARK-I and II) [47, 48]. On the basis of these trials, OPC is approved in the European Union, USA, Japan, Australia and other countries as adjunctive therapy to preparations of levodopa/DDCi in patients with PD and end-of-dose motor fluctuations [49] or OFF episodes [50]. A positive signal for OPC was observed on the Non-Motor Symptoms Scale (NMSS) miscellaneous domain, which includes pain, in both the BIPARK II trial [51] and the OPTIPARK study [52].

Given the probable dopamine-related pathophysiology of motor fluctuation-associated pain [12] and the encouraging signals detected in previous OPC studies, the OpiCapone Effect on motor fluctuations and pAiN (OCEAN) study has been designed. This trial aims to investigate the efficacy of OPC 50 mg in PD patients with end-of-dose motor fluctuations and associated pain, when administered as adjunctive therapy to existing treatment with levodopa/DDCi.

## Methods/design

### Study design

**Fig. 1:**
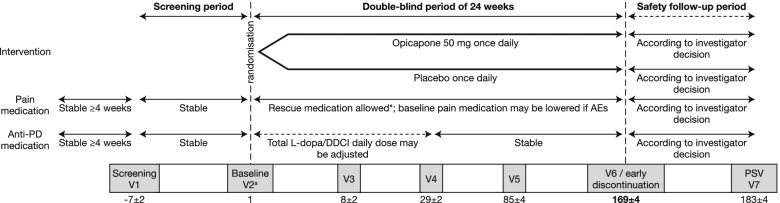
Study design. ^a^V2 is divided in V2a and V2b. If ON/OFF diary entries are non-compliant at V2a, the patient will be re-trained on correct use of the diary and visit V2b will be postponed for 3–4 days. If diary completion is satisfactory at V2a, V2b is performed immediately on the same day. AE, adverse event; DDCI, dopa decarboxylase inhibitor; L-dopa, levodopa; PD, Parkinson’s disease; PSV, post-study visit; V, visit

OCEAN is a Phase IV, international, multicentre, randomised, double-blind, placebo (PLC)-controlled, parallel-group, interventional trial in PD patients with end-of-dose motor fluctuations and associated pain (experienced for ≥4 weeks prior to the start of the study, with a score of ≥12 [out of 36] on Domain 3 of the KPPS at screening and baseline). It consists of a 1-week screening period, 24-week double-blind treatment period and 2-week follow-up period (Fig. 1). Following screening, at visit (V) 2 (baseline), eligible patients will be randomised 1:1 to OPC 50 mg or PLC once daily while continuing current treatment with levodopa/DDCi. Since OPC enhances the effects of levodopa, it may be necessary to reduce the patient’s levodopa/DDCi dosing within the first days or weeks of OPC treatment; therefore, the investigator may decrease the daily dose of levodopa/DDCi as needed until V4, while keeping the number of daily intakes unchanged. If necessary, dosing may be increased back to the baseline dose level. After V4, the levodopa/DDCi dose should not be changed until the end of the study. The anti-PD treatment regimen should be stable for at least 4 weeks prior to V1 (Table 2) and kept stable throughout the study (except for levodopa/DDCi during the adjustment period). No new anti-PD drugs should be started during the study.

**Table 2:** Inclusion and exclusion criteria

Category of characteristic	Inclusion criteria	Exclusion criteria
**Demographics**	• Male or female • Age ≥ 30 years^a^	
**Disease-related characteristics**	• Disease severity Stages I–III at ON^b^ • Signs of ‘wearing-off’ phenomena (end-of-dose fluctuations) with average total daily OFF time while awake ≥1.5 h (excluding early morning pre-first dose OFF period), despite optimal anti-PD therapy, according to the investigator’s judgment at V1 • At least 1.5 OFF h/day (excluding early morning pre-first dose OFF period), as recorded in the self-rated diary, during at least 2 of the 3 days prior to V2	• Non-idiopathic PD^c^ • Severe and/or unpredictable OFF periods (investigator’s judgment)
**Pain-related characteristics**	• Experiencing PD-associated pain for ≥4 weeks prior to V1 • Domain 3 of KPPS ≥12 at V1 and V2 • No changes in chronic treatment regimen for pain within 4 weeks prior to V1^d^	• Major/prominent non-PD-related pain (e.g. due to malignant disease)
**Anti-PD medication**	• Treated with 3–8 intakes/day of levodopa/DDCi^e^ and on a stable regimen for ≥4 weeks prior to V1 • Any other anti-PD medication regimen, if applicable, should remain stable for ≥4 weeks prior to V1 and should not be likely to require any adjustment until V6	• Treatment with prohibited medication^f^ within the 4 weeks prior to V1 • Treatment with apomorphine with 4 weeks prior to V1 or likely to be needed at any time until V6 • Previous or planned (during the entire study duration) levodopa/carbidopa intestinal gel infusion, deep brain stimulation or stereotactic surgery (e.g. pallidotomy, thalamotomy) • Previous or current use of OPC • Use of any other investigational product, currently or within 3 months (or five half-lives of the investigational product, whichever is longer) prior to V1
**Compliance**	• Adequate compliance with relevant PD and pain-related medication during the screening period (investigator’s judgment) at V2 • Filled in the self-rating diary in accordance with the diary instructions and with ≤3 missing entries/day in the 3 days prior to V2	
**Safety**	• Acceptable results of screening laboratory tests (i.e. not clinically relevant for the well-being of the patient or for the purpose of the study according to investigator’s judgment) at V2 • *For female patients:* postmenopausal for ≥2 years before V1, surgically sterile for ≥6 months before V1, or practicing effective contraception until V6^g^ • *For male patients:* use of condoms plus an approved method of highly effective contraception during the treatment period up to V6, if sexually active with a partner of childbearing potential	• Current or past (within previous year) history of suicidal ideation, suicide attempts or alcohol or substance abuse, excluding caffeine or nicotine • Pheochromocytoma, paraganglioma or other catecholamine-secreting neoplasms • Known hypersensitivity to the excipients of the investigation product^h^ or rescue medication • History of neuroleptic malignant syndrome or non-traumatic rhabdomyolysis • History of severe hepatic impairment^i^ • Previous history of psychosis or psychiatric disorders, including severe major depression • Any medical condition that might place the patient at increased risk or interfere with assessments • *For female patients:* pregnant or breastfeeding

^a^In Germany only, Age > 50 years

^b^Modified Hoehn and Yahr staging

^c^Atypical parkinsonism, secondary (acquired or symptomatic) parkinsonism, Parkinson-plus syndrome. “Idiopathic PD” was defined according to UK Parkinson’s Disease Society Brain Bank Clinical Diagnostic Criteria (2006) or Movement Disorder Society Clinical Diagnostic Criteria for Parkinson’s disease (2015)

^d^Includes medication (e.g. paracetamol, opioids, nonsteroidal anti-inflammatory drugs, antidepressants, anticonvulsants, corticosteroids) and non-medication therapies (e.g. transcutaneous electrical nerve stimulation, bioelectrical therapy)

^e^May include a slow-release formulation

^f^Entacapone, tolcapone, monoamine oxidase inhibitors (except selegiline up to 10 mg/day [oral] or 1.25 mg/day [buccal], rasagiline up to 1 mg/day, safinamide up to 100 mg/day) or antiemetics with anti-dopaminergic action (except domperidone)

^g^Female patients requesting to continue with oral contraceptives must be willing to additionally use non-hormonal methods of contraception during the course of the study

^h^Including lactose intolerance, galactose intolerance, Lapp lactase deficiency or glucose-galactose malabsorption

^i^Child-Pugh Class C

*DDCi* dopa decarboxylase inhibitor, *KPPS* King’s Parkinson’s Disease Pain Scale, *OPC* opicapone, *PD* Parkinson’s disease, *V* visit

Chronic pain treatment should be stable for at least 4 weeks prior to V1 (Table 2), and no new pain medication should be started during the study, except the allowed rescue medication (paracetamol or tramadol, based on the experience from the DOLORES trial [53]). The baseline dose of pain medication may be reduced during the study, if required due to pain medication-related adverse events (AEs), and increased again up to baseline dose level if necessary. Further visits will be performed on Day 85 ± 4 days (V5) and Day 169 ± 4 days (V6). The primary analysis will be performed on data collected at V6. A follow-up visit will be performed on Day 183 ± 4 days (V7), approximately 2 weeks after the last intake of study medication (OPC 50 mg or PLC). Patients who discontinue early will be requested to attend an early discontinuation visit. At V6 (or early discontinuation visit, if applicable), the investigator will arrange the patient’s subsequent treatment (i.e. either prescribe further OPC or switch to another treatment).

### Randomisation, blinding and allocation of treatment

At V1, each patient will be assigned in a chronological order via their electronic case report form to a unique patient number, which will be transferred by the site staff to an interactive web response system (IWRS). At V2b, after eligibility for entry into the treatment phase is confirmed, site staff will contact the IWRS to obtain the appropriate medication number. Randomisation will follow a 1:1 allocation rate (OPC 50 mg or PLC) and the randomisation list will be produced by the contract research organisation (Scope International AG, Mannheim, Germany) using the Statistical Analysis System (SAS) for Windows (SAS Institute Inc., Cary, NC, USA). The original list will be kept at the contract research organisation. Each patient’s investigational product will be determined by their randomisation number and corresponding medication number. OPC 50 mg and PLC capsules will be identical in size, colour, taste and appearance, and the packaging and labelling will not allow distinction between treatments. No person involved in conducting the study will have access to the randomisation code before the blind is officially broken. Unblinding will not occur unless there is an actual emergency and knowledge of the patient’s allocated treatment arm affects their treatment, in which case the individual treatment assignment for each patient will be available to the principal investigator/authorised delegate and responsible medical monitor via the IWRS. Patients with suspected unexpected serious adverse reactions (SUSARs) will be unblinded for regulatory reporting by the contract research organisation’s safety manager. Other study personnel and the investigators will receive blind information on the SUSAR until the study has been unblinded. The medication will be supplied by the sponsor (BIAL – Portela & Ca S.A., Coronado, Portugal) and the investigator/institution and/or a pharmacist or other appropriate individual (designated by the investigator/institution) will maintain records of delivery, inventory usage, and return any unused study medications. The investigator or an authorised delegate will be responsible for dispensing medication to the patients according to the dosage scheme and IWRS. At each study visit, site staff will dispense the appropriate amount of investigational product and rescue medication for each patient and for each treatment interval plus one extra week per 4 treatment weeks. At each visit, patients must bring back the study medication (including empty and partially empty containers) and accountability will be performed and documented.

### Ethical considerations

The study will be conducted in accordance with: the Declaration of Helsinki on Ethical Principles for Medical Research Involving Human Patients adopted by the General Assembly of the World Medical Association (2013); the applicable regulatory requirements of the participating countries; the International Council for Harmonisation of Technical Requirements for Pharmaceuticals for Human Use (ICH) Harmonised Guideline – integrated addendum to ICH E6(R1) Guideline for Good Clinical Practice E6(R2); and with the European Commission Directives 2001/20/EC and 2005/28/EC, and EU Regulation No. 536/2014. The protocol will be submitted to national Independent Ethics Committee(s) and Competent Authorities and unconditional approval/favourable opinion must be obtained before the start of the study. All patients must provide written informed consent in order to participate in the study.

Data processing will be conducted by the contract research organisation. This will include, but is not limited to, producing the patient diary and electronic case report form, and setting up a relevant database and data transfer mechanisms, along with appropriate validation of data and resolution of queries. Clinical data will be collected in electronic form using an electronic data capture system. All clinical data will be recorded, processed, handled and stored without disclosing personal information of the patients so that the data can be accurately reported, interpreted and verified while the confidentiality of records and the personal data of the patients remain protected, in accordance with the applicable rules on personal data protection.

### Study population

The study will be conducted in approximately 50 clinical sites in Germany, Italy, Portugal, Spain and the United Kingdom. Other countries and additional sites may be added, if required. Inclusion and exclusion criteria are outlined in Table 2.

### Study assessments

**Fig. 2:**
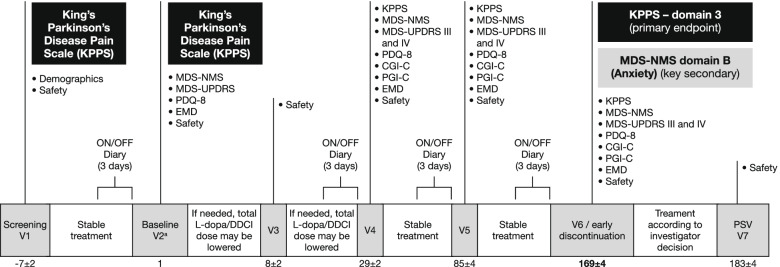
Timelines of study assessments. ^a^V2 is divided in V2a and V2b. If ON/OFF diary entries are non-compliant at V2a, the patient will be re-trained on correct use of the diary and visit V2b will be postponed for 3–4 days. If diary completion is satisfactory at V2a, V2b is performed immediately on the same day. CGI-C, Clinical Global Impression of Change; DDCI, dopa decarboxylase inhibitor; EMD, early morning dystonia; KPPS, King’s Parkinson’s Disease Pain Scale; L-dopa, levodopa; MDS-NMS, Movement Disorder Society-sponsored Non-Motor rating Scale; MDS-UPDRS, Movement Disorder Society-sponsored Unified Parkinson’s Disease Rating Scale; PDQ-8, 8-item Parkinson’s Disease Questionnaire; PGI-C, Patient’s Global Impression of Change; PSV, post-study visit; V, visit

An overview of study assessments is presented in Table 3 and the timing of these assessments is outlined in Fig. 2. Investigators will be trained on how to perform all assessments during each site initiation visit and at subsequent investigator meetings. Movement Disorder Society-sponsored Unified Parkinson’s Disease Rating Scale (MDS-UPDRS) training certificates will be provided to all sites according to MDS procedures.

**Table 3:** Overview of study assessments

Category	Assessment
Primary efficacy endpoint	Change from baseline in Domain 3 (fluctuation-related pain) of KPPS
Key secondary endpoint	Change from baseline in Domain B (anxiety) of MDS-NMS
Additional secondary endpoints	Change from baseline in Domain A (depression) of MDS-NMS
	Change from baseline in Domain K (sleep and wakefulness) of MDS-NMS
	Change from baseline in MDS-NMS total score
	Change from baseline in Domain 4 (nocturnal pain) of KPPS
	Change from baseline in KPPS total score
	Change from baseline in MDS-UPDRS Parts III and IV
	Change from baseline in PDQ-8
	CGI-C
	PGI-C
	Change from baseline in functional status via Hauser’s PD diary
	Changes from baseline in morning dystonia
	Use of rescue medication^a^
Safety assessments	Incidence of TEAEs, including serious TEAEs
	Changes from baseline in vital signs
	Changes from baseline in physical and neurological examinations
	Changes from baseline in routine laboratory parameters^b^

^a^Paracetamol or tramadol; ^b^haematology, serum biochemistry, pregnancy test

*CGI-C* Clinical Global Impression of Change, *KPPS* King’s Parkinson’s Disease Pain Scale, *MDS-NMS* Movement Disorder Society-sponsored Non-Motor rating Scale, *MDS-UPDRS* Movement Disorder Society-sponsored Unified Parkinson’s Disease Rating Scale, *PD* Parkinson’s disease, *PDQ-8* 8-item Parkinson’s Disease Questionnaire, *PGI-C* Patient’s Global Impression of Change, *TEAE* treatment-emergent adverse event

#### Efficacy

The primary efficacy endpoint is change from baseline in Domain 3 (fluctuation-related pain) of the KPPS. The KPPS evaluates the burden and characterises various phenotypes of pain in PD. It comprises seven domains including a total of 14 items. Each item is scored by severity (0–3) multiplied by frequency (0–4), resulting in subscores of 0–12. The total KPPS score (0–168) represents the symptomatic burden by pain [35].

The key secondary efficacy endpoint is change from baseline in Domain B (anxiety) of the Movement Disorder Society-sponsored Non-Motor rating Scale (MDS-NMS). The MDS-NMS comprises 13 domains covering a range of key PD- and treatment-related NMS, and a subscale for NMF that assesses changes in NMS in relation to timing of anti-PD medications across eight domains [54, 55]. Additional secondary efficacy endpoints comprise of other domains and total scores of the KPPS and MDS-NMS, change from baseline in the MDS-UPDRS Parts III and IV, change from baseline in the Parkinson’s Disease Questionnaire (PDQ-8), Clinical Global Impression of Change (CGI-C), Patient’s Global Impression of Change (PGI-C), change from baseline in functional status via Hauser’s PD diary, changes from baseline in morning dystonia, and use of rescue medication (Table 3).

The MDS-UPDRS is a revision of the UPDRS originally developed in the 1980s, and evaluates various aspects of PD; it consists of four parts [56]: Parts IA and IB, non-motor aspects of experiences of daily living; Part II, motor aspects of experiences of daily living; Part III, motor examination; and Part IV, motor complications. The PDQ-8 (a short form of the PDQ-39) is a patient-reported outcome that assesses eight aspects of functioning and well-being that are usually adversely affected by PD: mobility, activities of daily living, emotional well-being, stigma, social support, cognition, communication, and bodily discomfort. It rates overall health status by providing a single score ranging from 0 (good health) to 100 (poor health) [57]. The CGI-C and PGI-C are, respectively, investigator and patient assessments of how much a patient’s overall status has improved or worsened since the start of the study, comprising a 7-point scale: (1, ‘very much improved’; 2, ‘much improved’; 3, ‘minimally improved’; 4, ‘no change’; 5, ‘minimally worse’; 6, ‘much worse’; 7, ‘very much worse’). The Hauser’s PD diary is a patient record of their mobility during each 30-min period, categorised as: asleep; OFF time; ON time without dyskinesia; ON time with non-troublesome dyskinesia; or ON time with troublesome dyskinesia. When assessing changes from baseline in morning dystonia, the investigator will ask the patient if they experienced any morning dystonia within the last week (based on item 35 of the former UPDRS version). The amount and frequency of intake of rescue medication (paracetamol or tramadol) will be recorded by patients in a diary.

#### Safety assessments

Safety assessments include the incidence of treatment-emergent AEs (TEAEs), and changes from baseline in vital signs, physical and neurological examinations and routine laboratory parameters (Table 3; Fig. 2). At each study visit, the investigator will ask the patient in a non-leading manner about the state of their health in order to illicit information on TEAEs that may have occurred since the last visit. Any clinically significant observations made during the visit also constitute TEAEs. TEAEs will be documented as soon as possible in the electronic patient report form. The following information will also be specified: date/time of onset of TEAE; action taken with the investigational product; other actions taken; outcome of TEAE; seriousness of TEAE; severity of TEAE (mild, moderate, severe); and causal relationship of TEAE to investigational product (unrelated, unlikely, possible, probably, definite).

### Sample size calculation

For the primary efficacy endpoint (change from baseline in Domain 3 of KPPS), a difference to PLC of 3.0 is regarded as clinically meaningful. From a former study [53], a standard deviation (SD) of 5.8 can be assumed. With a two-sided significance α of 0.05, a power of 80%, a 1:1 treatment allocation ratio and with the above-mentioned assumptions, 2 × 60 = 120 evaluable patients are required. Assuming a drop-out rate of 15%, a total of 140 patients need to be randomised. Randomisation will follow a 1:1 allocation rate (OPC 50 mg or PLC).

### Statistical methodology

Efficacy assessments will be analysed for the Full Analysis Set, defined as all patients who are randomised and who have at least one measurement of the primary efficacy assessment. For sensitivity purposes, efficacy assessments will additionally be analysed for the Per-Protocol Set, defined as all patients included in the Full Analysis Set who have no major protocol deviations that could influence the primary efficacy assessment. The primary efficacy endpoint will be analysed using analysis of covariance (ANCOVA), with treatment as a fixed factor and baseline KPPS as a covariate, to demonstrate superiority of OPC 50 mg against PLC. Secondary efficacy endpoints will be analysed in an exploratory manner by treatment arm using appropriate parametric and non-parametric statistical methods. Descriptive statistics, including 95% confidence intervals, will be presented per treatment arm.

Safety assessments will be analysed for the Safety Set, defined as all patients who take at least one dose of investigational product. TEAEs will be summarised in terms of the number and percentages of patients with TEAEs. Vital signs and laboratory parameters will be summarised using summary statistics of absolute values and changes from baseline. Summary statistics and shift tables will be presented for physical and neurological examinations. Demographic and baseline characteristics will be presented using descriptive statistics. The statistical analysis plan will be carried out by biostatisticians from the contract research organisation.

### Current status

The first patient was enrolled in February 2021 in the UK. The recruitment window is now open and the last patient is expected to complete the study by late 2022. Timelines might be impacted by recurring COVID-19-related lockdowns impairing the access of patients to healthcare facilities.

## Discussion

Pain has a major impact on the quality of life of patients with PD [23, 58–60] and nociceptive pain accounts for the majority of reported pain in PD [22]. Since pain modulation involves striatal dopamine D_2_ receptors [61], pain associated with end-of-dose motor fluctuations may be alleviated through optimisation of dopaminergic therapy [12, 21, 22, 29].

Management of pain, among many other non-motor aspects of PD, remains a key unmet need [62] and there is currently a lack of robust data on the management of pain in PD patients with end-of-dose motor fluctuations. Previous studies in this setting have notable limitations, as well as varying both in the tools used to measure pain and the types of pain assessed. The Phase II PANDA trial was the first randomised controlled trial to specifically assess treatment for PD-associated pain [34]. Eligible patients were randomised to receive either prolonged-release oxycodone-naloxone or placebo. The types of pain patients experienced at baseline included musculoskeletal pain (73% in active arm), nocturnal pain (35%), fluctuation-related pain (32%) and PD-related chronic pain (26%). There was no significant difference between treatment arms in the average 24-h pain score at 16 weeks (primary endpoint). However, the measure used to assess pain was a general pain scale (a Likert scale) and levodopa was used more frequently as a rescue treatment in the placebo arm, both of which factors might have affected the results [34]. The double-blind, exploratory DOLORES trial was the first to investigate the effect of a dopamine agonist (rotigotine; administered as a transdermal patch) on PD-associated pain as primary outcome [53]. The types of pain patients experienced at baseline included musculoskeletal pain (51% in active arm), neuropathic pain (23%) and dystonic pain (14%). Although the findings suggested that rotigotine may improve PD-associated chronic pain in patients with advanced-stage PD, the trial was not powered to detect statistically significant treatment differences, due to the small sample size [53]. Safinamide (an agent with multiple modes of action, including monoamine oxidase-B inhibition) was shown to significantly reduce the need for pain medication, and to significantly improve two out of three PDQ-39 pain-related items, in comparison with placebo, when added to existing levodopa-based therapy [13]. However, these results were based on a *post-hoc* analysis of two previous trials and must therefore be interpreted with caution.

The design of the OCEAN study will address the current lack of reliable evidence for levodopa-based therapy in the treatment of PD-associated pain. OCEAN features recent validated PD pain- and non-motor-specific scales (such as the KPPS and the MDS-NMS), which might help to record dimensions of PD-associated pain that have not been previously studied. For instance, this study may allow the detection of potential associations between pain and other NMS, such as depression, anxiety and insomnia, and dysautonomic symptoms. The concomitant use of ON/OFF diaries with these scales may also allow a deeper understanding of pain and other NMS, such as the key secondary endpoint anxiety (as assessed by change from baseline in Domain B of the MDS-NMS), during both the OFF and ON states. Anxiety problems, including OFF-period anxiety, are highly prevalent in PD and greatly impact quality of life [63, 64]. Although some data suggest that anxiety symptoms inversely correlate with motor improvement induced by oral levodopa [65], this has been a neglected area in clinical investigation [30]. Placebo is known to activate dopamine receptors and to induce dopamine-like effects in PD [66–69], which are often still apparent in studies at 3 months [34, 47, 48], tending to wane by the 6-month mark [70, 71]. The 6-month course of OCEAN and its double-blind design might therefore help to disentangle the placebo dopamine-mediated effect from the true pharmacological benefit of OPC, especially when evaluating pain.

In summary, the OCEAN study will provide valuable information on whether the use of adjunctive OPC 50 mg treatment can improve fluctuation-associated pain in PD patients with end-of-dose motor fluctuations. The data will address the current lack of Level 1 evidence for the recommendation of strategies to manage aspects of pain in PD.

## Data Availability

Protocol details are available at www.clinicaltrialsregister.eu (EudraCT number 2020–001175-32). In line with EFPIA and PhRMA guiding principles, BIAL undertakes to share, upon request, anonymised patient-level, study-level clinical trial data (analysable data sets), and other information (such as protocols) from clinical trials in patients for medicines and indications approved in the United States (US) and the European Union (EU), to qualified researchers as necessary for conducting legitimate research.

## Abbreviations

AE: Adverse event
ANCOVA: Analysis of covariance
CGI-C: Clinical Global Impression of Change
COMT: Catechol-O-methyltransferase
COMTi: Catechol-O-methyltransferase inhibitor
DDC: Dopa decarboxylase
DDCi: Dopa decarboxylase inhibitor
EC: European Commission
ICH: International Council for Harmonisation of Technical Requirements for Pharmaceuticals for Human Use
IWRS: Interactive web response system
KPPS: King’s Parkinson’s Disease Pain Scale
L-dopa: Levodopa
MDS-NMS: Movement Disorder Society-sponsored Non-Motor rating Scale
MDS-UPDRS: Movement Disorder Society-sponsored Unified Parkinson’s Disease Rating Scale
NMF: Non-motor fluctuations
NMS: Non-motor symptoms
NMSS: Non-Motor Symptoms Scale
OCEAN: OpiCapone Effect on motor fluctuations and pAiN study
OPC: Opicapone
PD: Parkinson’s disease
PDQ-8: 8-item Parkinson’s Disease Questionnaire
PDQ-39: 39-item Parkinson’s Disease Questionnaire
PGI-C: Patient’s Global Impression of Change
PLC: Placebo
SAS: Statistical Analysis System
SD: Standard deviation
SUSAR: Suspected unexpected serious adverse reaction
TEAE: Treatment-emergent adverse event
UPDRS: Unified Parkinson’s Disease Rating Scale
USA: United States of America
V: Visit
